# Interactions at engineered graft–tissue interfaces: A review

**DOI:** 10.1063/5.0014519

**Published:** 2020-08-21

**Authors:** Wenzhen Zhu, Xiaolei Nie, Qi Tao, Hang Yao, Dong-An Wang

**Affiliations:** 1 School of Chemical and Biomedical Engineering, Nanyang Technological University, 70 Nanyang Drive, Singapore 637457; 2 School of Chemistry and Chemical Engineering, Yangzhou University, Yangzhou 225009, Jiangsu, People's Republic of China; 3 Department of Biomedical Engineering, City University of Hong Kong, 83 Tat Chee Avenue, Kowloon, Hong Kong; 4 Shenzhen Research Institute, City University of Hong Kong, Shenzhen 518057, People's Republic of China

## Abstract

The interactions at the graft–tissue interfaces are critical for the results of engraftments post-implantation. To improve the success rate of the implantations, as well as the quality of the patients' life, understanding the possible reactions between artificial materials and the host tissues is helpful in designing new generations of material-based grafts aiming at inducing specific responses from surrounding tissues for their own reparation and regeneration. To help researchers understand the complicated interactions that occur after implantations and to promote the development of better-designed grafts with improved biocompatibility and patient responses, in this review, the topics will be discussed from the basic reactions that occur chronologically at the graft–tissue interfaces after implantations to the existing and potential applications of the mechanisms of such reactions in designing of grafts. It offers a chance to bring up-to-date advances in the field and new strategies of controlling the graft–tissue interfaces.

## I. INTRODUCTION

During the designing processes of ideal implants for tissue engineering, the graft–tissue interface is the critical part to be studied all the time, since most of the interactions between the transplanted grafts and the surrounding host tissues take place around this area. The cellular and molecular levels of interactions at the interfaces are usually the critical determinants of the results of the transplantations macroscopically. Such interactions between living and non-living indeed are always causing confusion and problems during tissue engineering transplantations, such as bone absorption and desorption, blood clotting, fibrous encapsulation, etc. The problems keep occurring at the graft–tissue interfaces due to the fact that the “normal biology” becomes abnormal in direct contact with the foreign materials from the transplanted grafts *in vivo*. The so-called “intelligent surface” of the implants appears to be so static compared to the dynamic biology of the living organism. Specifically, an ideal tissue engineering implant is supposed to provide eligible surface chemistry that can incite particular cellular reactions from the surrounding host tissues, thus guiding new tissue to regenerate and prompting graft–tissue immerging. Although enormous effort has been devoted to studying the mechanism of the interactions that occurred at the graft–tissue interfaces to find out a certain technology to solve the problems, the jury is still out.

After transplantation, a designed implant is expected to trigger the healing and regenerating processes of the defective site *in vivo*, which are inevitably associated with inflammatory reactions.^1^ However, other unexpected reactions may be concurrent around the transplant sites depending on the implants' compositions, decompositions, or their surface chemistries.^2^ Over the years, various techniques have been used in the development of a desirable implant for tissue regeneration, such as chemical patterning,^3^ cell targeting,^4–6^ decellularization,^7–18^ polymer brush,^19^ and surface coating.^20,21^ At the same time, many medical technologies ranging from electrical sensors to drug delivery to medical implants^22–24^ have also been utilized to prompt the progression of this area.

The potential problems affecting the long-term performances and the research opportunities of a tissue-engineered product are unique. In recent decades, works on elucidating and controlling reactions at graft–tissue interfaces under different circumstances have been extensively studied and published. Many general biochemical processes such as protein absorption, cellular actions such as adhesion, and body reactions such as infection have been researched deeply, and many targeted methods have been used for graft–tissue products.^3,19–21^ Otherwise, some interaction has also been studied and been thought to be vital for the engineered-tissue graft effect, such as the rate of extracellular matrix (ECM) deposition, graft degradation, and bioactive surface-induced functional cell homing.^7,8,17,18,22–24^ But the mechanism and effective application in graft–tissue products are on their way. Getting inspired from these works, new ideas about designing novel biomaterial surfaces that are possible to integrate with the host tissues *in vivo* authentically are around the corner. The purpose of this review is to offer an overview of the interaction process at the engineered graft–tissue interface in aspects of biochemical, cellular, and system biological reactions and induced applications of upside process in biomaterials preparations.

## II. INTERACTIONS AT THE GRAFT–TISSUE INTERFACE

**FIG. 1.**
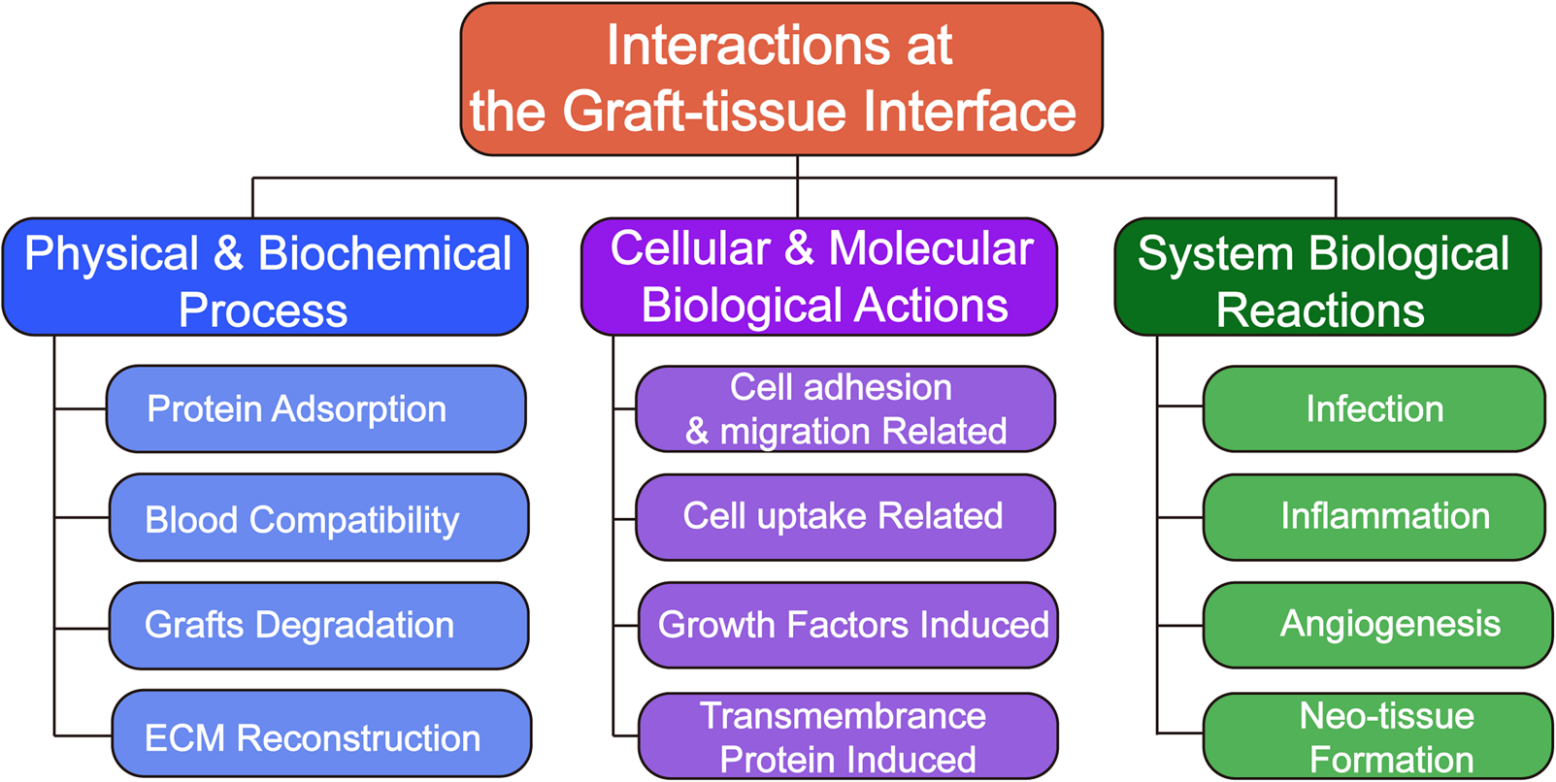
Schematic illustration showing the category of interactions at the graft–tissue interface.

As a “superorganism,” there are numerous chemical, electrical, or even mechanical actions and reactions occurring in the human body environment all the time.^25^ Similarly, a variety of biochemical actions and reactions occur dynamically at the graft–tissue interfaces.^26^ Since understanding the temporal progression of the chemistries starting from the first contact of biological molecules within an implant surface to the final tissue remodeling around an implant plays an essential role in understanding the tissue-implant interactions, in this part, the interactions at the graft–tissue would be introduced from three parts in different aspects: physical & biochemical process, cellular and molecular biological actions, and system biological reactions; the detailed structure is shown in Fig. 1. Many specific research points affiliated the three aspects such as graft degradation, cell migration, infection, and inflammation that would be introduced, which illustrate the process of the interactions, the effects on graft–tissue function, and the method of engineered product preparation.

### A. Physical and biochemical processes at the graft–tissue interfaces

The physical and biochemical processes are the basic and first founded and researched interactions of graft–tissue interfaces. The body fluid system and the vascular system are the places where the implants must interact when they enter the body, and so protein adsorption and blood compatibility have been part of that, which must be studied for the biocompatibility of the implant. Otherwise, based on the point of view of tissue engineering, biomaterials as the scaffold and cell or other bioactive things as the functional group are combined as a graft, It is hoped that the scaffolds degrade gradually, the new tissue or extracellular matrix (ECM) reconstructs sometime, and the functional part works all the time when the tissue has been grafted. Although the actual situation is not ideal upside, the ECM reconstruction and the graft degradation are vital process of grafted tissue interfaces and their one-to-one relationship sometimes determines the effect of engineered graft–tissue. Based on the above points, specific interaction aspects of protein absorption, blood compatibility, ECM reconstruction, and graft degradation would be introduced.

#### 1. Protein absorption at the interface

The affinity of a protein to different implant surfaces is affected by several factors, including its molecular size, hydrophilicity, and environmental pH.^27^ For example, an albumin molecule (67 kDa) could form significantly less contact with a silica substrate than fibrinogen (340 kDa).^28^ The hydrophilicity of a protein usually affects its affinity by the charge and the distribution of charges on the implants' surface, whereas the environmental pH can also affect proteins' affinity by affecting the surface charges of the implants. It has been reported that proteins tend to exhibit greater surface affinities if the environmental pH is approaching their isoelectric points.^29^

In addition, the folding or unfolding behaviors of a protein molecule can also affect its affinity since proteins always tend to expose more interaction sites for surface absorptions.^30^ The intramolecular cross-linking within a protein molecule is the main factor determining the extent or rate of unfolding,^31^ which is why less stable proteins or proteins with less intramolecular force would have greater surface activities. From the implants' side, the properties of biomaterial surfaces can also influence the interactions with proteins. The relevant properties include the implants' geometrical, chemical, and electrical properties.

For geometrical properties, it is reported that usually the more topographical features appear on the implant surfaces, the larger surface areas will be exposed for possible protein interactions.^32^

The implants' chemical properties affect the interactions with proteins mainly by determining the functional species available for the interactions. Specifically, metallic materials present metal and oxygen ions on the oxidized surfaces;^33^ ceramic materials present the metal and non-metal ions, while polymeric biomaterials present a wide range of functional species, including amino, carbonyl, carboxyl, and aromatic groups.^34^ Different species presented determine the affinity of the material to a certain group of biomolecules.^35^ It has been reported that hydrophobic surfaces have higher binding affinity to proteins.^36^ The electrical potential of the implants can affect the protein–material interactions by influencing the structure and composition of the surrounding electrolyte solution,^37^ as the electrical potential will rearrange water molecules and attract counterions whose combinatory effect can either enhance or hinder the interactions between proteins and biomaterial surfaces.^38^

It was learned from the above that many factors could affect and regulate the protein adsorption on the surface of the graft. It can be found that protein absorption is thought as the most basic process of graft–tissue interface interactions. Most of the biochemical and cellular processes begin from this, For example, it could accelerate thrombin and could form biofilm, some specific protein adsorption such as integrin could induce the target cell migration, and lubricin could make effort to the bio-lubrication. So an ideal protein adsorption surface design should base on the actual bioenvironment and specific applications. In general, it could be summarized in two aspects: From the biocompatible point of view, low protein adsorption hopes to be seen for the inert surface, which does not affect the origin biological process; from the functional application point of view, targeted protein adsorption hopes to be seen to achieve specific functional applications.

#### 2. Blood compatibility of the graft–material interface

During the procedures of implantation, bleeding is usually unavoidable. Therefore, blood is practically the first “tissue” that the surfaces of the implanted biomaterials will encounter. Blood is a multicomponent fluid containing over 150 kinds of proteins. Once in contact with a foreign surface, a variety of interactions can occur in between due to its complexity; according to the principle of mass transport, the proteins of highest concentration in the blood will be the first to arrive at the foreign surface. Thus, albumin, the most abundant protein in the blood, will arrive first. In combination with the moderate molecular size, albumin is frequently adopted as surface coatings or drug carriers to increase biocompatibilities of the implants.^39,40^ Nevertheless, although of a lower concentration and larger molecular size, immunoglobulin G (IgG) can still exchange with the albumin molecules bonded onto the foreign surfaces if allowing a longer time of reaction, which may be due to its significantly higher affinity to certain species over albumin.^41^ Following the same logic, other proteins still have the chance to replace the bonded molecules as long as they are processing higher affinity to the certain biomaterial. Thus, fibrinogen, for example, could overwhelm all other molecules, even though its rate of arrival is only less than one hundredth of that of albumin.^42^

Heparin, as a widely used surface coating compound, has been discovered to possess ignorable anticoagulant ability on its own, while its effectiveness can be manifested only in combination with antithrombin III, which is able to enhance its thrombin removal function.^43^ Heparin's anticoagulation mechanism hints that instead of being passively degraded, blood clots tend to be actively digested. When exceeding a certain threshold value, plasmin would initiate the fibrin destroying process, namely, fibrinolysis.^44–46^ At the coagulation site, the concentrations of various agents are controlled by staggering the releasing time-points of the agents, while the plasmin level will gradually rise over the course of 1–2 days after the initiation of fibrin digestion processes spontaneously.

Other than biomacromolecules, silver nanoparticles were found to have antimicrobial functions due to their high surface-to-volume ratio and continuous release of silver ions.^47–51^ Some other works have suggested that silver nanoparticles also have the ability to activate platelet via a physical collision mechanism and hence accelerate thrombin formation.^52–54^ Besides silver nanoparticles, several nano-scale modifications, such as high-density polymer brushes^55^ and nanowires,^56,57^ have been reported processing the abilities to minimize blood coagulation on implant surfaces. Even without additional coatings, studies have shown that high-density topography itself could help reduce thrombogenicity.^58,59^

#### 3. ECM reconstruction and graft-material degradation at the interface

As the milieu of cells in the body, the ECM is always taken as the objective to be imitated to improve the surface properties of implanted biomaterials.^60^ Since almost all the cell activities are performed by receiving complicated molecular and physical information from the ECM, and by interacting with the ECM, reconstructing extracellular stimuli in abridged forms is a useful way to manipulate cell fate, just like the reduction of biomacromolecules into short functional domains. Such techniques possess the advantage of easy-to-access and cost-effective. Usually, basic chemistry and biochemistry are the foundations to perform cell fate manipulation by ECM reconstructing.^61^

The concept of hydrolytically degradable biomaterial has been gradually established on the basis of previous research. Such biomaterial can be used as transitory implants to substitute or fulfill a certain function for a desired period of time, such as supporting healing progressions or controlling the release of drugs.^34^ These are the concepts analogous to absorbable sutures^62^ in surgical operations and implantable drug carriers used in cancer treatments.^63^ In both the scenarios, the degradability of the materials avoids a second around of surgical operations. Although the first use of the degradable surgical sutures and implants in clinical practice can be dated back to 40 years ago, today, a significant portion of doctors still prefers non-degradable sutures.^64^ One crucial reason is the degradation profile of the degradable surgical sutures, and implants are still unreliable and uncontrollable.^65^ Similarly, it is also a significant challenge for other implants made from hydrolytically degradable biomaterials.

In early degradable implants, lipids were often adopted as vesicles to control drug release,^39,66–69^ while hydroxyapatites were used to structure degradable bone implants.^70–72^ Besides those, polymers, especially biodegradable polymers, are a category of fundamental and extensively used materials in the development of degradable implants.^73^ These biodegradable polymers were mainly linear macromolecules composed of hydrolytically cleavable backbone bonds, such as ester and anhydride bonds.^74^

Although the implants for long-term applications are usually designed with non-degradable polymers, there is always a certain level of degradation or erosion *in vivo* since the *in vivo* environments are always more complicated than people's assumptions.^75^ Taking polyether urethane (PEU) as an example, although it is recognized as a non-degradable polymer, it has been reported that after implanting *in vivo,* the polymer would be oxidized by reactive oxygen species attached on the surface of the implants secreted by macrophages, and the resultant ester bonds could then be cleaved hydrolytically.^76,77^ The behavior of proteins at implants' surfaces is critical for defining the nature of graft–tissue interfaces. Because the proteins attached shall have impacts on the implants' surface properties,^78^ the surface properties of corresponding implants will, in turn, influence the adsorption of proteins on their surfaces.^79^

### B. Cellular and molecular biological reactions at the graft–tissue interfaces

A myriad of molecular and cellular reactions of breath-taking complexity have been involved in this progression. Inspired by human embryonic processes, the goal of tissue engineering is to regenerate or replace disabled or disordered human body parts by reconstructing some of the embryonic developments in miniature. So far, tissue engineering technologies have successfully treated a multitude of devastating diseases, including myocardial infarction,^80,81^ spinal injury,^82^ diabetes,^83^ and liver cirrhosis.^84,85^ Besides these achievements, nowadays, more and more attention has been transferred from traditional material design and engineering to advancing biomaterial applications for regenerative medicine. To make the biomaterials clinically available, the critical challenge to be overcome is the components of the designed formulas, which are too simple to influence the complicated cell behaviors *in vivo*. In addition, considering the costs and feasibility, an over-engineered implant would have little chance to be translated from research lab into clinical practices. At the current stage, compared to the ambitious goals of reconstructing the entire organs, more achievable goals have been set up to benefit patients to some degrees. For instance, clinical improvements in cardiac repairs converge on tissue engineering coronary arteries, valves, and myocardium rather than replacing the entire heart.^86–89^ Similarly, there has been a popular viewpoint in tissue engineering, saying that rather than endeavoring to reconstruct the complete complexity of living tissues *ex vivo*, designing and developing biomaterials that are able to establish pivotal interactions with host cells in ways that reveal the body's native organization and self-repair powers would be more achievable objectives.

#### 1. Cell adhesion and migration-related reactions

The ECM has been reported as a crucial body fluid participating in or even controlling numerous *in vivo* cellular events. That is why a number of scientific studies have been conducted to design and modulate ECM analogs, whose aim is to control cellular processes for regenerative medicine by ligating specific integrins.^18,90^ For example, fibronectin and its arginylglycylaspartic acid (RGD peptide) are widely used for cell adhesion.^91,92^ But the specificity was low due to the unselective engagement of its integrins. Recently, researchers have uncovered that the specificity of its integrins was affected by fibronectin's central cell-binding domains.^93–95^ Opposite to the enhancement of affinities, reduction of affinities is desired in some cases. CD47, a transmembrane protein, has been reported able to bind to signal-regulatory protein alpha (SIRPα) of leukocytes or macrophages and inhibit their attachments.^96–98^

Cell migration is a process in which cells move or proliferate along a certain chemical gradient or in a specific direction. When specific receptors on the cell membrane bind to chemicals existing in the surrounding environment, the cells will be stimulated to move in that general direction. Cell migration is dependent on cell adhesion. When the actin filaments in the cells are polymerized, the cells will bind tightly to the substrate underneath the elongated part of the cells. Subsequently, the actin filaments in the unstretched part will depolymerize and the adhesion bond between the unstretched cell body part and the substrate underneath will break.^99–102^ Overall, the cell will be moving toward the direction that the actin filaments are more frequently polymerized.

Mineral deposits have been reported to have the ability to cause accumulation of osteopontin, which, in turn, can augment cell adhesion and survival within phosphate-containing poly(ethylene glycol) (PEG) gels.^103^ Apart from showing the efficacy of mineral deposits in bone tissue engineering, new ideas in cell fate manipulations by chemical modifications have been put forward other than traditional understanding. Traditionally, chemical modifications tend to act directly on the cells. Nevertheless, it is also possible that the chemical modifications sequester cell-derived molecules providing behavioral signals to the cells.

Although scientists have attempted to design sophisticated chemotaxis on implant surfaces, cells can also alter the elaborate surfaces by producing new proteins to bind with or cover the ligands. To protect the engineered surfaces, a common approach is to co-graft the ligands with protein-resistant molecules. The so-called protein-resistant molecules are usually uncharged hydrophilic polymers, among which PEG is most frequently used. In addition, some other polymers have also been reported, such as carbohydrate-based coatings.^104,105^ These strategies have been reported to be helpful in temporarily exhibiting the desirable functions of the implant, but pieces of evidence had shown that the strategy might end up with failure when the amount of cell-derived proteins exceeded the capacity of the protein-resistant molecules.

#### 2. Cell uptake-related reactions

A living cell surface is a dynamic interface, not mentioning its convolution and heterogeneity. For example, cells keep detecting their environment by continuous pinocytotic processes, where cells swallow small particles suspended in the ECM surrounded, together with parts of the cell membrane from their own forming the vesicles. Every thirty minutes, the swallowed membrane parts can be internalized to keep the dynamic equilibrium of the cell's surface areas.^106^ Upon adhering to an implant's surface, cells begin to pull and rearrange the surface-bound biomolecules. Such reorganization plays a critical role in natural growth, yet commonly would not affect the molecules covalently bonded to the surfaces. Therefore, researchers have grafted ligands, such as RGD groups, to the material surfaces by non-covalent interactions in order to break the limitations of immobility so that the ligands can be transited to active clusters by cells.^107^

Although the covalently bound molecules are not able to be rearranged or reordered by cells, they can be modified by the cells by enzymatic cleavages. As an essential procedure *in vivo*, enzymatic cleavage paves the ways for cells to adjust the environment for preferable proliferation and migration. In the beginning, such modifications were regarded as adverse side effects for the well-designed implants since the delicately engineered protein coatings might be ruined shortly after transplantations. But later, the phenomena have promoted the replacement of protein coatings by peptide grafts as short polypeptides are more robust to proteolysis;^108–110^ on the other hand, they have also inspired the design and development of degradable materials. Unlike the hydrolytically degradable materials discussed in Sec. II A, these degradation processes are mediated by cells around the graft–tissue interfaces. For example, oligopeptides recognized by cell-secreted proteases, such as matrix metalloproteinase-2 (MMP-2) sensitive peptide, have been incorporated into functional biomaterials, such as PEG hydrogels, for tissue engineering applications.^111^

Commonly, phagocytosis processes are not applicable to implanted biomaterials due to the implants' large size. Therefore, after implantations, monocytes and macrophages would fuse and form multinucleated “foreign body giant cells,” which are usually found at the graft–tissue interfaces, in the attempt to phagocytose the large synthetic foreign bodies. These multinucleated giant cells (MNGCs) would persist for the duration of implants residing in the body of recipients.^112^ The above-mentioned reactions are the so-called “foreign body reaction.” It was also observed that textured material surfaces and surfaces with a high surface-to-volume ratio have the potential to attract more macrophages and MNGCs than smooth surfaces.^113–115^ On the contrary, smooth surfaces are more likely to induce denser fibrous capsules than rough or textured surfaces.^116–119^

#### 3. Growth factor and transmembrane protein-induced reactions

Growth factors are usually conjugated on the surface or encapsulated within the structure of the grafts. After implantations, the growth factors could be released into the interfaces between tissues and grafts following the degradation of their corresponding carriers. Growth factors are mediators released from cytoplasmic granules during activation and degranulation of relevant cells. They can have effects on cell migration randomly or along a growth factor concentration gradient, namely, chemotaxis. The objects of the growth factors can be *autocrine* or *paracrine*, while they can also contribute to cells existing in the surrounding tissues. For example, growth factors derived from activated platelets at the wound can diffuse rapidly from the blood circulation system into the surrounding tissues and body fluids to attract neutrophils, and subsequently monocytes, if the wound site is vascularized.^120,121^ These platelet-derived growth factors will be degraded in a short time by proteases through normal biochemical processes after inducing those attracted cells to secrete subsequent growth factors.

Cells can receive signals by contact with other cells, interacting with the ECM, and exposing to locally released growth factors to help activating and/or inducing the expression of their own cell-membrane receptors. During these processes, transmembrane proteins are the bridges between the extracellular environment and the intracellular cytoskeleton proteins. Integrin receptors affect the formation and disruption of the focal adhesions, which are critical for the cell migration processes. Bindings of integrin receptors to ligands can trigger a series of intracellular chemical events, which will subsequently be passed on to the cell nucleus.^122,123^ This type of signaling is termed “outside-in” signaling, where the intracellular signaling is initiated through integrin receptors binding to an extracellular ligand. On the other hand, there is also “inside-out” signaling, where a cell can regulate its extracellular milieu by altering either cytoskeleton conformation or integrin receptor expression/activation. Some of the transmembrane-initiated pathways indeed overlap with the pathways used by growth factors.

### C. System biological reactions at the graft–tissue interfaces

The engineered-tissue graft is the product of biomedical engineering, which is judged by the effect and the function in the end. The clinical problem is to test their standards, which were the same as the system biological reactions when the graft has been used. The problems of infections or antibacterial and inflammation were the common effect and most concerned issue in clinical surgery. Otherwise, when we talk about the tissue regeneration, four factors need to be concerned: mechanical support, angiogenesis, nerve regeneration, and functional regeneration. Therefore, this part would overview from aspects of infection, inflammation, angiogenesis, and functional regeneration to illustrate the reactions at the graft–tissue interfaces.

#### 1. Infection responses and antibacteria

The infection problem is the most common issue in clinical surgery. Infection is mainly due to the proliferation of bacteria, which causes the body's immune response. The graft–tissue interface was a place for bacteria to multiply for the biofilm formation.^124^ Based on current research and reports, the formation of biofilms on the implant surface mainly comes from the following aspects. The structure of the implant itself is limited, and more extreme sterilization strategies cannot be used, which makes the surface of the implant itself carry bacteria. In addition, the implant does not have a targeted antibacterial strategy, and the biofilm will be easily formed on the surface of the implant.^125,126^ Second, the surface of the implant is not treated with special anti-protein adsorption treatment, which makes a large amount of body fluid protein aggregate on the surface of the implant. At the same time, the antibacterial measures of the implantation operation are not strict, which causes the formation of biofilms.^127^

From the study of the above-mentioned biofilm production process, it is not difficult to find that the functional antibacterial mechanism and the anti-non-specific protein adsorption on the inert surface of the implant are important means to reduce the infection of the implant. In terms of active antibacterial, peptide antibacterial, nanoparticle antibacterial, antibiotic carrier type surface coating, and other methods based on antibacterial engineering methods have been used in many antibacterial strategies for implants.^128–131^ However, there are relatively few studies on active antibacterial in shaping the environment for anti-biofilm formation. Early engineered implants have more inert surfaces. Researchers think more and try to make the surface absolutely inert, with very little adsorbed protein to achieve the effect. But in fact, in a complex, long-term internal environment, absolute inertia is more difficult to achieve. Therefore, at present, some researchers have directly changed their thinking to directly modify functional proteins on the surface of the implant, and after implantation, they can compete with the biofilm formation environment to achieve specific functions and achieve results.^132,133^

#### 2. Inflammation reactions

Acute and chronic inflammations always occur following the initial blood-material interactions. In acute inflammatory responses, neutrophils are the dominating cells.^134–136^ Histamine released by the neutrophils, together with fibrinogen, are adsorbed by mast cells as the mediators to acute inflammatory responses, after which interleukin-4 and interleukin-13 released by mast cells regulate the progress of foreign body reactions.^137^ The histamine may further transform into phagocyte chemoattractant,^138^ while the fibrinogen adsorbed on the implant surfaces will facilitate the phagocyte adhesion. Usually, acute inflammatory responses would resolve within one week post-implantation.^139,140^

The inflammatory response is a trigger of systemic biological responses, one of whose beneficial consequences is vascularization. The inflammatory phase may start to be resolved with the rise of blood vessels around the implants.^141^ Specifically, on the appearance of blood vessels, ECM components will be synthesized and deposited around the graft–tissue interfaces, followed by the sequential presences of proteoglycans and collagens. The neo-capillaries will make the tissue appear granular since the presence of proteoglycans and collagens is closely correlated with the chronological development of the granulation tissue.^142^ The granulation tissue is the histologic indication of normal resolution of the inflammatory phase.^143,144^

#### 3. Tissue regeneration and angiogenesis

In a long-term view, it is the regenerative capacity of various cell populations that determines the outcome of the damaged tissue repair by implantations.^145,146^ The cells can mainly be categorized into three different types in terms of their regenerative capacities, which are labile cells, stable cells, and static cells. Labile cells, such as epithelial, lymphoid, and hematopoietic cells, keep proliferating throughout life; Stable cells, such as vascular endothelial cells, chondrocytes, osteoblasts, smooth muscle cells, and fibroblasts, are capable of proliferating but only proliferate in response to suitable stimuli, whereas static cells (or permanent cells), such as nerve, skeletal and cardiac muscle cells, cannot reproduce after birth. Therefore, theoretically, tissue regeneration can only be expected to occur in tissues containing labile or stable cells.

To systemically integrate the implants to the organism and sustain the neo-tissue formation, vascularization is crucial. Angiogenesis relies on the presence of the ECM and endothelial cell migration.^147–149^ Some vascularization stimulating growth factors [mainly fibroblast growth factor (FGF) and vascular endothelial growth factor (VEGF)] released in hypoxic and slightly acidic environments by different cell types have been reported to be able to promote the growth of capillaries in the neo-tissues.^150^ These new vessels will continuously deliver oxygen and nutrients via circulating blood.

Angiogenesis is a complex process involving the proliferation and organization of endothelial cells into blood vessels. Therefore, besides angiogenic growth factors, matching the ligands existing in the ECM to the surface receptors on the endothelial cell membrane is crucial for proper cell migration and other pertinent processes. It has been observed that the vascular restenosis caused by the competitive adhesion between endothelial cells (ECs) and smooth muscle cells could be a transition from angiogenesis to scar generation.^151–153^ It has also been observed that after the defect or wound site is healed and filled with neo-tissue, some of the newly generated blood vessels tend to disintegrate as a result of apoptosis. Such a phenomenon is currently believed to be regulated by the ECM.^154,155^ Specifically, after the damaged tissue being substantially replaced by neo-tissue, the cellularity of the neo-tissue would, in turn, become lower.^73^ The exact triggering mechanism of this cell apoptosis remains to be further elucidated. Simultaneous with neo-blood vessel disintegration, synthesis and degradation of the collagens would also be altered, including the relative amount of different collagen types specific to different tissue types and the structure of collagen fibrils, such as the thickness and orientation. It has also been observed that, even after the defect site is completely refilled by self-regenerated neo-tissue without complication, the resulting new tissue may not able to replicate the functions of the original tissue.^156^ In other words, self-healing or self-regeneration may lead to scar tissue formation.

Pieces of evidence have shown that biochemical factors, for instance, high levels of transforming growth factor-β and mechanical stresses at the wound sites, may contribute to scar formation.^157,158^ The main differences between scar tissue and normal tissue are the relative amount, types, and structure of collagens.^159^ In scar tissues, all the collagen fibrils are arranged as parallel bundles, such an arrangement has been proved mechanically to be inferior to normal tissues and prone to re-injury. So far, the underlying mechanisms of scar formation are not fully understood. The desired outcome after implantation of grafts is timely resolution of the wound healing process and achieving a steady state, in other words, terminating the wound healing-related biological changes and integrating the neo-tissue to the surrounding biological milieu without fibrous capsule formation.

## III. DESIGN IMPROVEMENT OF IMPLANT PERFORMANCES

The function of cells, and eventually the structure of the tissues built by the cells, can be regulated by the surrounding environment, which indeed is the ECM containing large numbers of nano- to micro-scale signals. However, the cells often respond erroneously in the presence of synthetic materials designed for tissue engineering disease or injury treatments. It is a challenge for researchers to integrate the native signals into tissue-engineered biomaterials to regulate cell-material interactions.

Based on this review in the second part of the reaction process, phenomenon, and mechanism at different levels of the implant surface interface, it is not difficult to find that the physical properties, chemical activity, and biological activity of the implant surface can affect the implant surface interface reaction. However, based on specific implant applications, the realization of highly characteristic and targeted implant surface interface reactions is the fundamental purpose of the specific application of physical, chemical, and biologically active substance modification. Therefore, in this part, the thesis will introduce different research studies using these physical and chemical modifications to design functional implant materials for the surface interface reaction of the implant (Table I).

**TABLE I.** Methods of design improvements for functional implant performance.

Modification category		Methods of design improvements for functional implant performance	Mechanism	Applications
Morphology	Surface roughness construction	Different porosity	Increased specific surface area	Cell adhesion improvements^172,173^
		Oriented surface topology	Physical barrier	Tissue formation with a forward structure and^170^ cell migration^165^
	Construct regular three-dimensional structures	Porous structure	More live spaces	Cell adhesion and proliferation improvements and cell living space arrangements^146^
		Grid structure	Physical barrier in 3D	
		Layer-by-layer construction	Bionic structure design	Specific neo-tissue formation improvements^148^
Physical and chemical properties	Surface energy change	Plasma treatment	Surface functional group changes	Changes in the adsorption capacity of proteins and active molecules and cell surface protein stimulation^38^
		Surface potential adjusting	Surface potential change	
	Surface wetting properties	Hydrophilic treatment	Hydrophilic changes	Cell attachment regulation, interface micro-environment control, and cytokine adsorption^175^
		Hydrophobic treatment		
	Others	Molecular brush modification	Bionic structure design	Implant intergration^177^
		Inorganic particle spraying	Bioactive group function	Bone-related tissue formation^33,162^
		Functional carrier particle coating	Drug carrier mechanism	Functional molecular delivery and control release^39,66–69^
Biochemical properties	Functional peptide chain coating	RGD	Cell protein interaction	Cell adhesion and migration improvements and cell morphology control^183,184^
		Fibronectin		
	Functional protein coating	Collagen	Cell protein interaction	ECM reconstruction and cell adhesion improvements^186^
		Specific antibody	Immune function	Specific cell interactions and signal pathway regulation^185^
	Surface bioactivity group graft		Bioactive group function	Enable complex or specific biochemical interactions^179,182^

### A. Morphological modifications

Researchers have been trying to influence cell and tissue responses by modifying surface roughness and morphology of the implants. The most widely studied approaches are the alterations of surface porosity of the implants achieved by coatings. Such alterations have been reported to be beneficial for native tissue ingrowth, which will, in turn, facilitate fixations of the implants due to mechanical interlockings at graft–tissue interfaces. This type of coating is most extensively used on orthopedic implants, such as hip and knee replacements, currently many of which have been well-studied and commercialized. Other applications taking advantage of this approach include anchoring of heart valve cuffs into heart muscles as well as penetration of percutaneous implants into the skin.^160^ Moreover, studies have shown that more patterned morphologies, such as a grooved surface, can better guide cell orientation and migration to desired directions. This mechanism has been adopted to prevent epithelial down growth in dental implants^161^ and to direct bone^162^ and blood vessel formations^163,164^ along specific regions of an implant.

In order to ultimately rehabilitate the graft into functional tissues, large numbers of infiltrated cell populations and adequate nutrient deliveries are important in augmenting the success rate of cell survival and growth. Studies have shown that porous structures with connected conduits are good for effective and sufficient transport of nutrients, oxygen, and metabolic waste in and out of the implants. Such structures can be produced using various techniques including induced phase separation techniques, freeze-drying, particle leaching, and sintering. However, rather than focusing on the surface of bulk materials, the most often reported technique of porous scaffold fabrications for tissue engineering is electrospinning. Xie *et al.* reported a centrifugally aligned poly(ε-caprolactone) nanofiber presenting nanoscale topographic cues to promote cell proliferation and migration from the edge to the core.^165^ It was found that the type I collagen secreted by the cells cultured on these scaffolds demonstrated organized alignment.

Rapid prototyping is a relatively novel technique, which is able to sequentially deposit programmed tomographic surfaces layer-by-layer to build a three-dimensional implant. This technique offers researchers the opportunity to mimic the heterogeneity of the pore sizes more accurately. Using the rapid prototyping technique, the variation of pore sizes from trabecular to cortical bones has been successfully reproduced with polycaprolactone (PCL).^166^

Surface roughness mainly affects cell adhesions and fibrous encapsulations. Miller *et al.* have treated the surfaces of poly(lactic-co-glycolic acid) (PLGA) with sodium hydroxide to increase the surface roughness on the nanoscale.^167^ Recently, the osteogenic differentiation of mesenchymal stem cells (MSCs) has been demonstrated to be impacted by surface roughness. PCL substrates with roughness varying in the range of micrometers and higher peak densities were shown to promote MSC osteogenic differentiation.^168,169^ In addition, titanium surfaces with nano-scaled surface roughness have shown a similar osteogenic differentiation promoting effect on MSCs.^170,171^

Moreover, titanium with increased surface roughness has also been reported to have stronger macrophage spreading ability. Specifically, sandblasted and acid-etched surfaces seemed to have higher possibilities to elicit the secretion of inflammatory cytokines by macrophages.^172,173^

### B. Physicochemical modifications

Physicochemical modifications include alterations in surface energies, surface charges, and surface compositions. Glow discharge is a mainstream method to increase surface free energies of metals and polymers. During the process, the surfaces will be exposed to ionized inert gases, which can break bonds and cross-link polymeric materials. As a result, the surface permeability will be decreased, while the surface hardness increased. Due to the critical role of electrostatic interactions in many biological events, surface charges are believed to be able to affect protein and cell behaviors at graft–tissue interfaces. Grafting acidic or sulfonate-containing functional groups can achieve negatively charged surfaces, whereas amino-containing functional groups are often used to produce positively charged surfaces. Negative charges can delay thrombogenesis, while positive charges can accelerate it. Furthermore, cationic particles are more likely to aggravate inflammatory responses than anionic or neutral species, which is believed to be owing to the natively negative charged immune cell membranes. Specifically, the positive charges on the graft surfaces can neutralize the negative surface charges on the cell membranes, thereby inducing signal transductions into the cytoplasm and stimulating inflammatory reactions. Specifically, mixing of polypyrroles, an electrically conductive material with ECM components, can achieve sequential myogenic differentiation of primary myoblasts.^174^ This could be due to the mimicking of the native physiological environment. It can be an inspiration for material designers in inducing the recovery of tissues composing electrically responsive cells such as neural and muscle cells.

Besides surface charge, surface wettability can also affect the biological behaviors of immune cells. Normally, hydrophobic materials tend to increase monocyte adhesion compared to hydrophilic materials, causing local immune reactions *in situ*. It has been reported that hydrophilic/neutral copolymer surfaces can inhibit macrophage adhesion.^175^ But the cells successfully adhered to the surfaces would secrete larger amounts of cytokines and chemokines than those on hydrophobic or hydrophilic surfaces.

Compared to surface energy and surface charge, surface chemistry can always bring more dramatic changes to the materials. It can be achieved by grafting macromolecules onto biomaterial surfaces. Two typical examples are the self-assembled monolayers (SAMs) and polymer brushes. SAMs of alkanethiols on gold substrates have been reported to be able to avoid nonspecific protein adsorption *in vitro* and can be further modified with non-covalently bonded ligands to control *in vitro* cell adhesion, ECM assembly, and cell differentiation.^176^ Nonetheless, since the *in vivo* environment is quite different from the *in vitro* setup, this model system suffers from lacking stability *in vivo*. To overcome the drawback of the SAM-based model system, polymer brushes were exploited, which has been revealed to be more robust on diverse biologically relevant substrates. Furthermore, titanium implants have been coated with poly(oligo(ethylene glycol)methacrylate) brushes in order to equip them the ability to tether bioactive ligands and, therefore, to facilitate the implants' integrations *in vivo*.^177^

Other than synthetic polymers, naturally derived biomolecules have also been incorporated into implants. Resolvin D1, a pro-resolution lipid mediator, has been adopted into porous 3D chitosan-based scaffolds. In this case, the scaffolds were empowered with immunomodulating effects and able to initiate a transfer in macrophages toward an M2 reparative response.^178^ Moreover, oestradiol, an estrogen steroid hormone and one of the main female sex hormones, was loaded in poly-l-lactic acid (PLA) electrospinning fibers, which enhanced the integrations of the polymeric mesh into host tissues by stimulating new blood vessel formations.^179^ This tissue-engineered material was reported to have the potential in treating weakened pelvic floor problems in women.

Other than biomolecules, inorganic molecules were extensively studied for potential applications in the bone tissue engineering field as one of the main compositions of bone is a crystal-form inorganic bone mineral. Comprehensive research has been conducted on PLA electrospinning nanofibers coated by hydroxyapatite, bioactive glasses, and tricalcium phosphate particles.^180^ The results of the *in vivo* studies showed that all the three kinds of coatings achieved a high calcium content in the reconstructed bones. Moreover, hydroxyapatite bioactive glass-coated nanofibers showed even higher efficiency in osseointegration than the other combinations, which could be used as a promising bone graft in treatments of orthopedic fractures and defects.

### C. Biochemical modifications

Biological surface modifications are based on molecular biology principles, whose design philosophy is to regulate cell and tissue reactions to a specific implant by grafting biomolecules onto its constructional materials. The most widely used cell attachment promoting molecule is the RGD sequence. Various lengths and conformations of the sequence have been tested to verify its selectivity on specific integrins. Another frequently-used peptide is heparin/heparan sulfate-binding peptides, which are used in combinations with the RGD sequence to further promote cell adhesions. Ting *et al.* reported a modified chitosan surface that was crosslinked by genipin and subsequently treated with heparin. It was found that UE7T-13 cells were able to quickly proliferate on the modified fibers; yet, red blood cells could hardly attach to the fibers.^181^ This type of fiber mesh showed strong potential to be used as vascular gaskets. A comparison between chemical and biological modifications has also been conducted using sulfonated Polyethylene oxide (PEO) and heparin; however, no significant difference was concluded between these two types of modifications.^182^

Rather than using simple peptide fragments, intact protein molecules and other biomacromolecules have been deposited onto the surfaces with the objectives of equipping multiple functions to the implants by various domains within the molecules. Growth factors, for example, have the potential to induce cell growth, activity, and differentiation simultaneously. On the other hand, biological surface modifications can also be used to prevent cell adhesions. Phosphorylcholine has been grafted onto or incorporated into biomaterials to imitate phospholipid heads of cell membranes, thus repelling other cells to avoid unexpected cell anchorages.^183^

Due to the intensive research studies on the functions of biological modifications to implants, a head-on comparison of different biomacromolecules would be essential. A comprehensive study on collagen, chitosan, and Gly-RGD-Ser peptide-decorated PCL electrospinning fibrous scaffolds has been conducted and revealed that type I collagen, integral protein-containing RGD sequences, showed the highest capability to improve both the attachment and the proliferation of fibroblasts, keratinocytes, and pre-osteoblastic cells.^184^

Different from general cell attachment and adhesion, selective cell attachment is critical for vascular grafts since the unfavorable consequences, namely, thrombosis, and the favorable consequences, namely, endothelialization, will occur simultaneously post-engraftment. Therefore, specific antibodies have been adopted as surface biological modifications. Lu *et al.* reported a heparin/collagen multilayer coating containing anti-CD133 antibody on polytetrafluoroethylene (ePTFE) graft, which showed a prolonged blood coagulation time and prevented platelet activation and aggregation but promoted endothelial cell adhesion.^185^

Despite the special proteins adopted in the ePTFE graft, the ECM is always the key source for researchers to search for useful proteins. Various proteins have been extracted from the ECM, including collagen I, III, IV, laminin, fibronectin, etc. Therefore, the ECM has often been directly used as the biomaterial or been coated on other synthetic materials to enhance the implants' integration with native tissues and to prevent fibrous encapsulation. Holger *et al.* have extensively studied the reactions of different organisms to the ECM by subcutaneously implanting decellularized ureters as model implants in rats. From the study, fibroblasts and M2 anti-inflammatory macrophages were found to be the primarily infiltrated cells.^186^

Since the outcomes of tissue regeneration are largely dependent on the releasing time and dosages of growth factors, instead of simply presenting growth factors on implant surfaces, controlled release of these molecules has also been investigated. For instance, in bone lesion repairs, collagen sponges containing recombinant human bone morphogenetic protein-2 (BMP-2) are clinically accessible for degenerative disk treatments. Due to the short *in vivo* biological half-lives of the above-mentioned growth factor, sustained release and short diffusion distance between a bound growth factor and a targeted cell are vital to maximize the effect of the growth factor.

## IV. CONCLUSION AND PERSPECTIVES

Graft–tissue interface reactions can be affected by a variety of factors, which, in turn, leads to different features and tissue repair efficacies of the implant. Even for a single graft–tissue interface combination, the reactions will be quite different in different anatomic locations. Since the upsurging of tissue engineering, numerous biomimetic scaffolds have been developed using synthetic and natural-derived materials. As the field has grown, more questions have emerged. For example, what is the minimum level of materials' complexity required for activating endogenous or transplanted cells to initiate generating a complex tissue? Furthermore, based on current reports, biochemical reactions, cell biological behaviors, and system biological responses should be clear. Through specific biomedical engineering methods, a system with specific interface response orientations should be constructed. The surface interface response is controllable, and it is developing toward the functional applications we need, which leads to an ideal implant surface. Therefore, designing dynamic systems started to emerge as a new research theme. Moreover, tissue ingrowth is affected by the pore size and shape of the porous implants. Hence, nanofabrication and fast prototyping methods, such as three-dimensional printing, have been developed as a promising alternative method to control such factors compared to traditional methods more precisely.

Today, more and more researchers are trying to pre-seed cell populations into the implants. Such cells could be syngeneic, allogeneic, or even xenogeneic, which could cause unique issues to host–graft interactions. After implantations, the tissue-engineered grafts might be subjected to inflammatory cytokines, growth factors, and ECM, which are different from the original “simple” environment *in vitro*. This “compromised” environment could evoke variable responses, bringing about a myriad of challenges. One of the feasible methods to meet such challenges is cell-free biological grafts, which could achieve the desired bioactivities of the graft and avoid the destructive responses of the cells post-implantation at the same time. For example, rather than implanting grafts with pre-seeded living cells, the cells could be pre-cultured on the scaffolds for ECM depositions and subsequently removed by decellularization methods, leading to a cell-free ECM-based graft. In another instance, rather than transplanting MSCs, whose main role is currently believed to be paracrine effects, the cells could be cultured *in vitro*, and only the paracrine factors can be harvested for the following treatments. Although such types of implants are still in the emerging stage, due to their enormous potential to be applied clinically, it could be a promising direction for future research.

Some other challenges related to graft–tissue interactions include inducing abundant vascularizations, industrializing established implants, and accurate controlling the degradation rates for different applications. Future implants for tissue regeneration applications will possibly exploit more functionalization techniques or surface modifications for the sake of enhanced bioactivities than banking on the substantial properties of the unmodified biomaterials themselves. Furthermore, with the increase in human life expectancy, there will be a growing demand for long-term implants. The implants have the ability to induce and enhance the self-healing mechanism of human bodies, will definitely become the research focus of tissue engineering in the future. We hope that explaining both the tissue and implant schemes in the common language of chemistry and biochemistry at the interfaces will be supportive for future research.

## Data Availability

Being a Review Article, data sharing is not applicable to this article as no new data were created or analyzed in this study.
